# School-life and Physical Development1President’s address delivered before the 26th annual meeting of the Medical Association of Central New York, Held at Rochester, N. Y., June 16th.

**Published:** 1893-10

**Authors:** Louis A. Weigel

**Affiliations:** Rochester, N. Y.; President of the Medical Association of Central New York; 43 Mortimer Street


Buffalo Medical and Surgical Journal

Vol. XXXIII. OCTOBER, 1893. No. 3.

### Original Address.

### SCHOOL-LIFE AND PHYSICAL DEVELOPMENT.[1]

By LOUIS A. WEIGEL, M. D., Rochester, N. Y.

President of the Medical Association of Central New York.

It is quite the proper thing at the present day to indulge in physical culture. The merchant, the professional man, the clerk and the artisan feels it his duty to atone for sins against his personality by exercise.

Well-equipped gymnasia and facilities for athletic sports of various kinds are at his command, which enable him to indulge in physical culture according to his taste and fancy. Learned dissertations upon the necessity of exercise to overcome the direful effects of our enervating mode of life exist in profusion; and in perusing them one is led to believe that in exercise alone is to be found the universal remedy for the violations of the laws of Nature. We have no desire to underestimate the good that may be accomplished by properly regulated exercise; at the same time, it may not be out of place to give a little consideration to natural physical development in contradistinction to the artificial.

It goes without saying that the early years of life are preëminently the ones in which the body may and should be developed in a natural way. It is also the period during which proper physical development is most easily interfered with. During the very early periods of life, it may be assumed that Nature is usually allowed to have her sway, and is unhampered in her efforts to gradually bring about a nicely adjusted coördination of the functions of the body. But as the child of Nature advances in years, the time for artificial education arrives, which means that it must be subjected to conditions entirely at variance with its former habits of life, and

1. President's address delivered before the 26th annual meeting of the Medical Association of Central New York, held at Rochester, N. Y., June 16th.

which may or may not exert a deleterious influence upon its physical development, according to circumstances.

In taking up the question of school-life and its effect upon physical development, we are confronted with a difficult task, for it is not an easy matter to determine to what extent premature or close mental application is responsible for defective bodily vigor. Investigations instituted with this object in view, must of necessity be general in character, and cannot take into account the individual, however desirable this might be. Our schools are made up of children in all stations of life, and thus represent the average of a given community. It is by a study of this average that we are enabled to arrive at some conclusion with reference to the perfection or imperfection of a system of education, in so far as it affects the health of children.

It is said that "our modern system of education imposes too great demands upon the young organism in the critical period of its growth; that it seeks too one-sidedly to stimulate mental growth, and that the physical development is thereby so neglected that great dangers arise to the body, as well as to the closely related mental health." Let us briefly examine these charges against our school system.

It is obvious that statistics with reference to the health and physical development of school children, to be of any value, must include a large number. Investigations of this character have been made in several foreign countries and the results published. Thus, in 1881, a fundamental research was instituted in Copenhagen, and its result was so significant that a special hygienic commission was appointed to examine the conditions of health in all the schools of the country. This commission has examined nearly 15,000 boys from the middle or preparatory schools, and 3,000 girls in private schools, in reference to their health, and has measured and weighed them. The results of these researches show that boys pass through three different periods of growth: First, a moderate increase in their seventh and eighth years; second, a weaker growth from the ninth to the thirteenth years, and a much more rapid increase in height and weight from their fourteenth to sixteenth years, or during the period of puberty. The growth continues after the last period, but more slowly.

The development of girls, also, presents distinct periods, but the changes occur a few years younger than in boys. Comparing the subject by stations in life, it was found that the more rapid growth begins a year earlier in the children of well-to-do classes than in those of the poorer classes. Scanty and hard conditions of life are restrictive and hindering to the growth of children. The growth of the poorer children previous to puberty is prolonged at the cost of the latter. It is as if something hindered these children from entering their period of more rapid development in the same year of life as the children living in better circumstances. The development of puberty is delayed in them, but as soon as it is begun it goes on with increased rapidity, and in spite of the delay, is completed in the same year as it is in better situated children.

It is an interesting question, and especially important in relation to education, whether the growth of children goes on evenly during the different seasons in summer and winter. Researches show that children exhibit a relatively light growth from the end of November to the end of March. This period, which includes all the winter months, is followed by a second, from the end of March until July or August, during which children grow rapidly in height, but their increase in weight is reduced to a minimum. After this follows a third period continuing to the end of November, in which the increase in height is very small, but the gain in weight very large; the daily accession of weight is often three times as great as during the winter months.

Investigations by this same commission among school boys as to the percentage of illness, demonstrated the fact that during the period of weak growth, which precedes the coming on of puberty, and during which pupils are passing through the preparatory or low classes of the schools, the power of resistance of the youthful organisms against external influence is diminished.

During the period of development of puberty, on the other hand, when the youthful life is approaching maturity with all its swelling force, the capacity for resistance rises from year to year, the liability to illness falls, reaching its minimum in the last year of that period. Immediately afterwards sets in another period of diminished capacity for resistance, which usually includes the last years of school-life; the very time when the greatest demands are made upon the pupil.

The percentage of illness among girls is even greater than among boys, as it was found that sixty-one per cent. of the whole, all belonging to the well-to-do classes, were ill or affected with serious chronic disorders. Such a condition of affairs, growing worse in the years preceding puberty and during its beginning, while it is not materially improved in the last years of the period, certainly deserves careful attention.

Professor Key, of Stockholm, in commenting upon this, says: "The amount of work, sitting still, etc., exacted of the girl, is not consistent with her health during her growing time. Without going into particulars as to the influences injurious to the health of growing children, which proceed from their homes, or may be brought out in connection with the school and school-work, it is still manifest that the burden of work that children have to bear under the present school regulations far exceeds what is permissible, and is, to a large extent, responsible for the liability of school children to illness."

It may be objected that statistics regarding the condition of children in some foreign countries do not apply to American boys and girls; that in this country the social surroundings and general mode of life are better. We are said to be better fed and better housed. Be this as it may, it cannot be denied that the nervous strain to which children are subjected in this country, both in and out of school, more than counterbalances our boasted advantages in other directions.

In this country no extensive researches have been made to show the relation of school-life to growth and health. Most of the investigations I am familiar with were made with the object of obtaining data for special purposes.

I should suggest that physicians, particularly in the larger communities, institute inquiries in a systematic and thorough manner, so that, eventually, we may be able to determine in what manner physical development and growth during the critical periods of childhood and youth are influenced by school-life. Similar researches in rural districts would be valuable for a comparative study of the influence of city and country life upon children during the years they attend school.

It would also be of interest to extend our inquiries to children in reformatories and other institutions, where the daily routine of life is, perhaps, painfully regular. In reformatories we would find a class of children who grow up under less restraint, and in whom the natural propensities and impulses have been to a larger extent unchecked. We would probably find that good physical development is the rule.

In collecting data, the special points to be considered are age, sex, weight, rate of growth as between boys and girls of the same age, rate of growth at different periods of school-life, the percentage of illness, and its nature, whether functional or organic, etc., etc. By collecting observations bearing upon these points, we will eventually be able to pass judgment upon the merits and value of certain systems of education, and to determine to what extent the health of children is affected by them.

As Professor Key says: "It is incumbent on us to see with all possible care that the growth of youth during their years of puberty, which is so full of importance, is not disturbed or distorted by any influence adverse to Nature. But as instruction is now arranged, at school and at home, we should first of all direct attention to the phase of a child's age immediately preceding the period of puberty, when the growth is at its lowest, the child's capacity for resistance is weak, and his liability to illness increases from year to year. We must learn how to obviate this liability to illness, and it is for science to forge the weapons with which to do it."

It is not within the scope of this paper to dilate upon the various diseases and malformation for which the exactions of the school-room are responsible; you are all familiar with them. It may not be out of place, however, to say a few words upon the subject of prophylaxis. As already stated, there exists a popular notion that physical exercise is a universal remedy, applicable to the child as well as to the adult. Our educators seem to realize that the health of children is affected by long-continued mental application, and attempt to provide relaxation by the introduction of physical exercise, in the shape of calisthenics, or some other gymnastics. But such exercises, as usually practised, fall far short of what is actually required, and may be mildly termed a farce. Is it not true that the very children who need physical exercise the most, take the least interest in what is intended for their physical betterment? This is, perhaps, due to the fact that the methods adopted are not sufficiently recreative in character, or are too difficult of execution. As Lagrange well says: "It is obvious that difficult exercises cannot be recreative. This is still a great reproach to our gymnastics, when we undertake to apply it to children subjected to school-work, and who have so great need of amusement and distraction in the intervals between their studies. It is not a relaxation that the brain of a child can find in these methodical exercises, but, rather, one lesson more added to so many others. Among the movements of our gymnastics, those which are not hard enough to discourage the child by long apprenticeship, are so destitute of interest that they repel by their monotony. Such, for example, are the 'floor' exercises. Forty children, ranged in three lines, wait, with erect body and fixed eye, the command of the master. Then, all together, at his order, turn the head first to the right, then to the left; they count aloud, one, two, three; and, while counting, extend their arms, bend them, raise them, drop them; then the legs have their turn, and, finally, the trunk and loins. All these motions are very hygienic, but where is there a place for transport and joy in that cold discipline, that fixes the features and effaces the smile, in those insipid gestures, of which the slightest distraction would destroy the grouping. Yet, to the pupil, pleasure is not only a moral satisfaction; it is a hygienic element, indispensable to his health. To impose on a child exercises in which he will find no pleasure, is more than a want of solicitude—it is an offense against hygiene." Furthermore, he says: "Our artificial methods of gymnastics are not favorable to the physical education of children, because they are athletic, and not hygienic, methods. They look especially for strong subjects to make champions of them, when a good hygiene should look for weak subjects to make strong ones of them." We must not forget that the weak form a large majority of the children of the present generation. Our children, so precocious now in their mental development, are far behind in their bodily growth. They need methods of education adapted to their weak physical aptitudes. This is the capital fault of artificial and difficult methods. They do not bring exercise within the reach of children. They are, properly speaking, methods of "selection." They subject children to a sort of trial, taking the strongest to make athletes of them, but leaving the weakest, or the great majority, delivered to all the physical and moral woes that re derived from want of exercise.

Artificial and difficult exercises are to natural exercises what, in mental education, the higher instruction is to primary and secondary instruction. Physical education has its "grades," as well as mental education, and we commit an error when we reverse them.

We do not, perhaps, sufficiently appreciate the true relation between the muscular and nervous systems. Long ago, Dubois-Reymond reminded us of the fact that all bodily exercises are really exercises of the central nervous system, the brain and spinal cord. It is true, a certain amount of muscular power is necessary for exercise, but this is not all. A man may have the muscles of a Hercules, and still be unable to stand or walk, to say nothing of executing more complicated movements. For instance, simple intoxication is sufficient to deprive him of the power to coördinate his movements properly. Every action of our bodies, as a locomotive apparatus, depends more upon a correct coördination of the muscles than upon the strength of their contraction. The real mechanism must be located in the central nervous system; consequently, exercise of muscles is essentially an exercise of the nervous system. A harmonious development of these two systems is necessary, for, as Maudsley says: "He who is incapable of guiding his muscles is incapable of concentrating his mind."

Recognizing the applicability of artificial gymnastics, especially the machine variety, to children, it may be a question whether it is desirable or wise for our boards of education to add gymnasiums, with their elaborate appliances, as a part of a school outfit, or to engage teachers of gymnastics, who are an expensive and unnecessary luxury. It would be no great loss to dispense even with the æsthetic Delsarte system. We are already provided with a perfect gymnasium. We have plenty of lawns, shady streets, public parks, etc., where children may indulge in gymnastics best suited to them, that is, natural, unrestrained movement. You will, no doubt, agree with me that a half-hour's unrestrained exercise in the open air accomplishes more good than any amount of artificial work.

In all natural movements we use a large number of muscles at once, and we sometimes bring into action those which are very remote from the point where the work appears to be localized. Active games constantly tend to the division of the work among a large number of muscles. This is the consequence of the very character of natural exercise. Being copied from instinctive acts, of which they are simply the methodical regulation, they will present the same character of causing the human machine to execute much work without demanding much effort from it. The hygienic quality of exercise is not effort, but rather work. The more work we do, the more we stimulate the great vital functions, notably, respiration, and the circulation of the blood.

There is one more point to which I call your attention. In order to indulge in physical exercise, either in the shape of gymnastics or games, a certain amount of muscular power is necessary. Now, occasionally we find a child so exhausted, both physically and mentally, that it is utterly impossible for it to make any muscular effort. Its nervous system has been subjected to such a degree of tension, that any form of exercise would simply throw an additional burden upon it, and add fuel to the fire. Such children are never at rest, not even during sleep; they toss about the whole night, and arise in the morning exhausted, instead of refreshed. They have indulged in involuntary muscular exercise at a time when Nature intended them to be at rest. In such cases, a mode of life which would place the child, to a certain extent, in a passive condition, would be far more beneficial than enforced activity.

Our American method of living is one of ceaseless activity, and the time may come when we will be forced to acquire the ability to rest, which we certainly do not have now.

In briefly presenting these points, I intended them to be suggestive in character. You all realize that the subject is a prolific one, and one upon which there is, perhaps, a great deal of misconception. By making investigations, as indicated, we may be better able to fit the demands of the child's organization to its strength and capacity of resistance during the different periods of his growth; better able, than we are now, to devise means to promote his health and physical development. One hundred years ago, the author of school hygiene introduced his warning against a too early and too sudden strain upon the physical powers of the mind and body, with these words: "Yet, spare their fibers, spare their minds' strength; waste not upon the child the vigor of the man that is to be."

43 Mortimer Street.

---

Migraine.—Migraine may be relieved, Lucking says, with a pill, twice daily for some time, consisting of Indian hemp one-sixth grain, phosphate of zinc one-tenth grain, and arsenic one-thirtieth grain. The severity of the attack may be effectually diminished with liquor trinitrine, in minim doses, two or three times daily.—*N. Y. Med. Record.*

